# From solution to structure: empowering inclusive cryo-EM with a pre-characterization pipeline for biological samples

**DOI:** 10.1107/S1600576724001717

**Published:** 2024-03-26

**Authors:** Christoph Mueller-Dieckmann, Alessandro Grinzato, Grégory Effantin, Daphna Fenel, David Flot, Guy Schoehn, Gordon Leonard, Eaazhisai Kandiah

**Affiliations:** a Structural Biology, European Synchrotron Radiation Facility (ESRF), 71 Avenue des Martyrs, Grenoble 38000, France; b Univ. Grenoble Alpes, CNRS, CEA, IBS, Grenoble 38000, France; Oak Ridge National Laboratory, USA; North Carolina State University, USA

**Keywords:** cryo-electron microscopy, cryo-EM, single-particle analysis, vitrification, cryo-electron tomography, SOS pipeline

## Abstract

In addressing the challenges faced by laboratories and universities with limited (or no) cryo-electron microscopy (cryo-EM) infrastructure, the ESRF, in collaboration with the Grenoble Institute for Structural Biology (IBS), has implemented the cryo-EM Solution-to-Structure (SOS) pipeline. This inclusive process, spanning grid preparation to high-resolution data collection, covers single-particle analysis and cryo-electron tomography (cryo-ET). Accessible through a rolling access route, proposals undergo scientific merit and technical feasibility evaluations. Stringent feasibility criteria demand robust evidence of sample homogeneity. Two distinct entry points are offered: users can either submit purified protein samples for comprehensive processing or initiate the pipeline with already vitrified cryo-EM grids. The SOS pipeline integrates negative stain imaging (exclusive to protein samples) as a first quality step, followed by cryo-EM grid preparation, grid screening and preliminary data collection for single-particle analysis, or only the first two steps for cryo-ET. In both cases, if the screening steps are successfully completed, high-resolution data collection will be carried out using a Titan Krios microscope equipped with a latest-generation direct electron counting detector coupled to an energy filter. The SOS pipeline thus emerges as a comprehensive and efficient solution, further democratizing access to cryo-EM research.

## Abstract 2

The European Synchrotron’s Solution-to-Structure (SOS) pipeline streamlines cryo-EM studies, providing an inclusive process from grid preparation to high-resolution data collection for single-particle analysis and cryo-electron tomography. This service is especially tailored for users with restricted or no access to cryo-EM infrastructure, enabling them to conduct screening experiments and acquire samples suitable for high-resolution data collection.

## 1. Solution-to-Structure (SOS) pipeline

Single-particle analysis (SPA) and cryo-electron tomography (cryo-ET) have become routine structural tools to resolve high-resolution three-dimensional structures of biological macromolecules (Ignatiou *et al.*, 2024 ▸). While cryo-electron microscopy (cryo-EM) facilities hosting one or several high-end microscopes are being established worldwide and smaller institutes are investing in auto-loader-equipped 200 kV electron microscopes, cryo-EM experiments still remain unreachable for many (smaller) laboratories and even universities. The current access model to apply for experimental time on high-end microscopes, such as the CM01 (Titan Krios) operated at the European Synchrotron (Kandiah *et al.*, 2019 ▸), for SPA is through the presentation of both an established cryogenic protocol to prepare sample grids and a prior (smaller) data collection showing 2D classes for single-particle analyses, justifying the use of this state-of-the art microscope (Chua *et al.*, 2022 ▸). This access model has proved very useful for the evaluation process, ensuring the efficient use of high-end microscopes. However, a weakness is that – by definition – it excludes projects from groups that have limited or no access either to intermediate microscopes to perform screening and pre-characterization or to sample grid preparation robotics. A few leading cryo-EM centers in the United States, such as the Pacific Northwest Cryo-EM Center and the Stanford-SLAC Cryo-EM Center, and a few EM facilities in Europe (INSTRUCT centers) offer services for grid preparation and screening for academic users. However, these services are not adequate to address the needs of the wider user community. Recognizing this gap, the ESRF has implemented an extension, called the Solution-to-Structure (SOS) pipeline, of the cryo-EM services it offers to external users. For SPA, this pipeline includes sample grid preparation, grid screening and collection of a small data set to evaluate the quality of obtainable data and, if justified, the collection of a full data set using a high-end microscope. Additionally, for cryo-ET, the SOS pipeline combines sample grid preparation and grid screening, facilitating the collection of full tomography data when deemed appropriate.

### 1.1. Workflow organization of SOS pipeline

**Figure 1:**
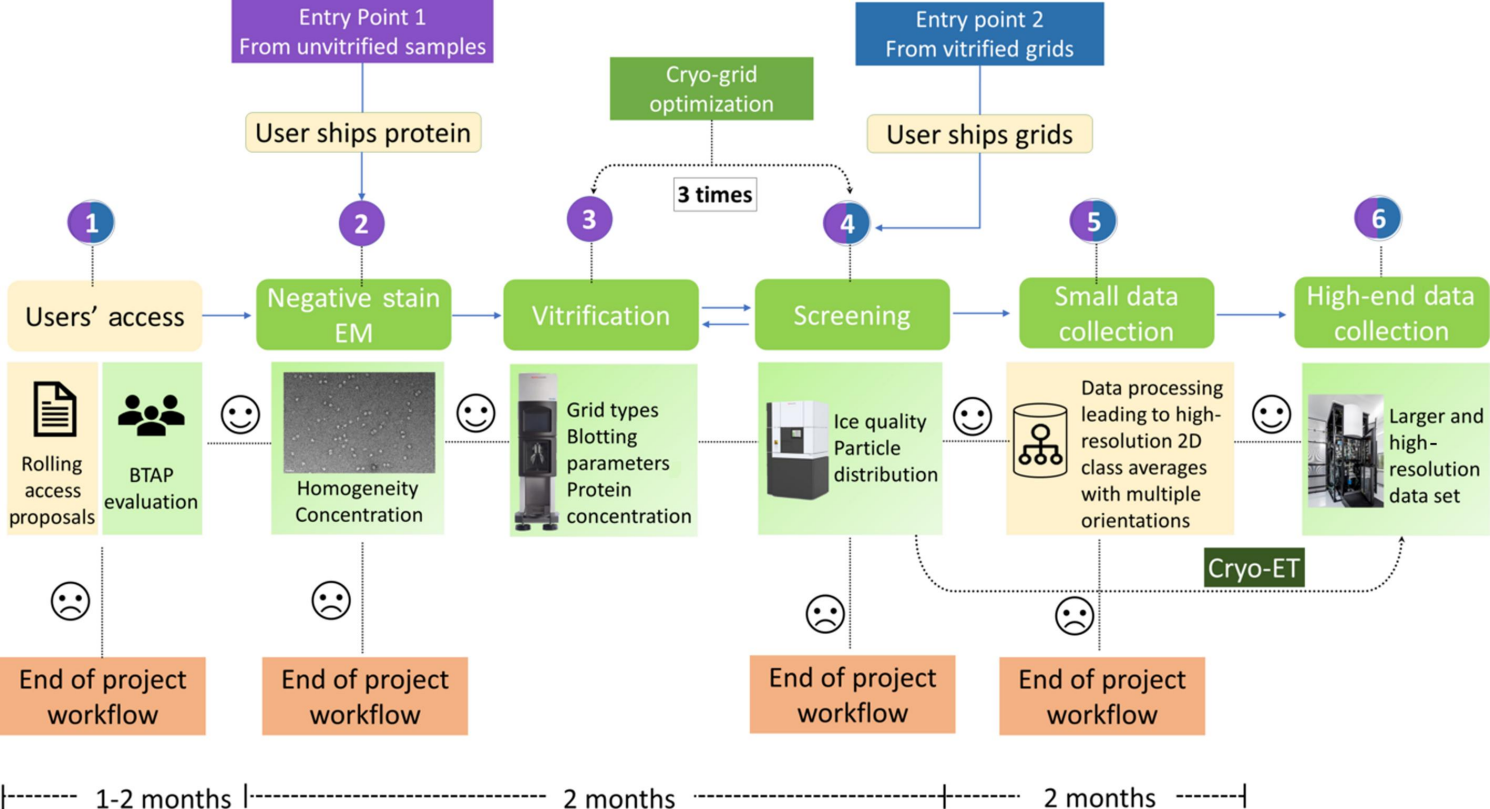
The workflow organization of the SOS pipeline is illustrated, emphasizing the tailored nature of the pipeline for different sample types and showcasing two distinct entry points: one originating from unvitrified samples, highlighted in magenta, and the other from vitrified grids, denoted in blue. Cellular samples for cryo-ET are not subjected to the NS-EM step. Common steps, applicable to both entry points, are depicted, along with specific procedures unique to each. Notably, Step 5 is omitted for cryo-ET experiments. The actions performed by users are indicated by boxes colored in beige.

To access the SOS pipeline, users are requested to write a project proposal (Step 1). This is rapidly evaluated by ESRF’s macromolecular crystallography (MX) Beamtime Allocation Review Panel (BTAP), composed of external specialists in the field of structural biology, with information being kept confidential. If access is granted, the pipeline offers two potential entry points (Fig. 1 ▸).

For the first entry point, users send their purified protein samples to the ESRF, which then will be processed using the following steps:

Step 2: quality control using negative staining EM (NS-EM). Subsequent steps are executed only if Step 2 is successful.

Step 3: preparation of cryo-EM grids using a Vitrobot.

Step 4: grid screening using a Glacios microscope (located at the IBS, Grenoble).

Step 5: collection of a ‘small’ data set in the case of a successful Step 4 (only for SPA).

Step 6: high-end data collection using a Krios microscope located on the ESRF CM01 beamline.

The second entry point of the pipeline enables users to start with already vitrified cryo-EM grids. In this case, Steps 2 and 3 above are skipped and the pipeline will start with Step 4 (Fig. 1 ▸). In this scenario, users are required to furnish quality control NS-EM images obtained at their home institute. In both cases, all steps are carried out by scientists/technicians from the ESRF and the IBS.

#### 1.1.1. ESRF access mode for SOS pipeline

To ensure optimal use of this pipeline, the following boundary conditions have been established. Currently, only bio-safety level 1 samples are accepted. Samples will be considered technically feasible only when accompanied by strong supporting biophysical evidence demonstrating their homogeneity (Chua *et al.*, 2022 ▸). Such evidence should include the results of analyses conducted through techniques such as sodium dodecyl sulfate polyacrylamide gel electrophoresis (SDS-PAGE), size-exclusion chromatography (SEC) and other biophysical characterizations, such as SEC coupled with multi-angle light scattering (SEC-MALS) or small-angle X-ray scattering (SAXS), together with confirmation of the buffer’s suitability for cryo-EM.

The SOS pipeline is accessible to all ESRF users who meet the defined feasibility criteria. Access requests can be made through the rolling access route (https://www.esrf.fr/UsersAndScience/UserGuide/Applying/ProposalGuidelines/MXRollingCryoEM). Proposals can be submitted at any time throughout the year and will undergo a comprehensive evalua­tion process, commencing with a scientific merit assessment by the ESRF’s MX BTAP and a technical feasibility evaluation conducted by CM01 scientists.

It is important to highlight the strengths and specific conditions of the SOS pipeline. For instance, as described earlier, to increase the chances of success, users will be asked to provide proof of stability from various biophysical studies. If the protein seems stable only in buffer conditions containing over 4% glycerol or high concentrations of sugar, our beamline scientists are available for consultation to ensure experimental success. The SOS pipeline is particularly well suited for analyzing macromolecules with a molecular mass exceeding 75 kDa, given the complications arising from low contrast associated with smaller sized molecules. In the context of cryo-ET experiments, the pipeline accepts both non-cellular samples and cellular samples that do not require sectioning, such as mini-cells. A maximum of three vitrification trials and cryo-grid screenings are included in the pipeline, with only the latter applicable for entry point 2.

The cryo-EM grid preparation and optimization, including all the consumables and beamtime for screening and data collection, are provided to users at no cost. Users may choose to participate either on site or remotely during the grid preparation and screening stages to provide scientific input to help the process. Travel expenses are the responsibility of users. However, for the final step of Krios data collection, considered a standard rolling access experiment, ESRF covers users’ travel expenses. Additionally, due to the demanding nature of this experiment and the substantial scientific expertise required, co-authorship has currently become a common agreement, underscoring the collaborative spirit of our research community.

#### 1.1.2. Negative stain imaging for cryo-EM feasibility

For an accepted proposal starting with purified protein samples, Step 2 is dedicated to NS-EM and serves as a critical checkpoint to assess the quality of the sample for cryo-EM. For cryo-ET samples that are not suitable for NS-EM such as cellular samples, this step is excluded. This step is performed as part of a service using the IBS-EM platform (https://www.isbg.fr/samples-preparation/quality-control-by-electron/). Under this service, various NS-EM grid conditions (*e.g.* various stains, protein concentration *etc.*) are exhaustively explored to confirm the sample’s homogeneity and the best starting point for moving to Step 3. Samples displaying a high degree of aggregation, insufficient concentration or substantial heterogeneity in NS-EM are considered unsuitable for cryo-EM, resulting in the end of the proposal workflow.

#### 1.1.3. Cryo-EM grid preparation

Following a successful completion of Step 2, the subsequent Step 3 involves vitrification using a Vitrobot IV grid preparation robot. The first attempt comprises the preparation of eight grids using various grid types [*e.g.* Quantifoil (Cu) or Ultrafoil (Au)] and employing a basic set of Vitrobot blotting parameters such as blot time and blot force applied to two different protein concentrations. If necessary, graphene-coated grids and surfactants can be tested.

#### 1.1.4. Cryo-EM grid screening

With the aim of ensuring the quality of the ice and the particle distribution for high-resolution data collection, the vitrified grids are subject to a subsequent examination using a Glacios microscope (Thermo­fisher Scientific Inc.), managed by the IBS-EM platform (Step 4). This initial screening provides valuable data concerning parameters such as ice thickness and particle distribution and will reveal suitable and sustainable areas for data acquisition. Subsequently, a second vitrification trial is carried out with the intent of fine-tuning these parameters. This entails a variation of grid type, blotting parameters and protein concentration, followed by another round of screening using the Glacios microscope.

Both Step 3 and Step 4 allow a maximum of three rounds of optimization each. In the event that no suitable grids are found within this allotted number of trials, the research project is regrettably discontinued. Users are promptly notified by a written report of the outcome, which can serve as a valuable guide for optimizing their sample. That may involve the inclusion of supplementary partner proteins (Uchański *et al.*, 2020 ▸; Grisshammer, 2017 ▸) or the implementation of crosslinking stabilization techniques (Weissenberger *et al.*, 2021 ▸; Schmidt & Urlaub, 2017 ▸). The report also enables users to make improvements and subsequently to reapply with an optimized sample for further experiments.

#### 1.1.5. Preliminary data collection to assess the quality for high-resolution potential for SPA

This step is exclusive to SPA and is not performed for cryo-ET. In the event that favorable grid conditions are obtained in the screening rounds, a small data set is acquired using the Glacios microscope equipped with a K2 direct electron detector (Step 5). The data will be stored in the ESRF’s central storage system according to the ESRF data policy (https://www.esrf.fr/datapolicy) and will be accessible to users through the ESRF’s transfer protocol (Kandiah *et al.*, 2019 ▸), with appropriate access permissions granted for data transfer. The data will remain stored in their current location for 90 days from the experiment date. They will also be archived on tape for long-term storage. Access to data on tape will be available to both users and the public through the ESRF’s data portal, with users having continuous access and the public gaining access after a three year embargo period (https://www.esrf.fr/datapolicy). Users are entrusted with the task of processing this data set to furnish the ESRF with the requisite feedback, which serves as a basis for evaluating the feasibility of high-resolution data collection and, subsequently, the justification for full data collection on a Titan Krios microscope (Thermofisher Scientific Inc.) (Step 6). In order to achieve this, users are asked to show, in their original application, the availability of adequate resources and expertise in single-particle image processing.

#### 1.1.6. High-resolution data collection on Krios

SPA users should provide feedback on the preliminary data collected using the Glacios equipped with a K2 camera within a reasonable delay. If the 2D classification results imply several views and averages of sub-10 Å resolution, either the same grid used for the Glacios data collection or one from the same batch will be considered for high-resolution data collection using the Titan Krios. Conversely, if image processing fails to reveal high-resolution details in the 2D classification, the proposal will be concluded at this stage.

For cryo-ET, once suitable grids are obtained in Step 4, the experiment will be scheduled for high-resolution tomography data collection.

### 1.2. Timeframe of SOS pipeline

The initial stages of proposal processing for the SOS pipeline closely resemble the standard procedures applied to rolling access proposals, with a typical timeframe ranging from one to two months. Subsequent steps, from NS-EM to cryo-EM sample preparation trials, extend the time by approximately two months. If the cryo-EM sample demonstrates favorable grid conditions, a limited data set of around 2000 micrographs is acquired using a 200 kV Glacios microscope equipped with a K2 direct electron detector. User feedback on image processing of this data set is anticipated within the following two months, during which time the selected grids are securely stored at the ESRF. This phase is pivotal for ensuring grid quality for high-resolution data collection. The seamless integration of these stages streamlines the process, making it efficient and effective for researchers utilizing the SOS pipeline.

## 2. Perspectives

The overarching goal of the SOS pipeline is to make cryo-EM research more accessible to a broader user community. Researchers from member countries such as Italy, India and Portugal have successfully utilized the SOS pipeline to achieve high-resolution structures, starting from liquid protein samples (Zarzecka *et al.*, 2020 ▸). There is also a growing demand to initiate processes from vitrified grids, and interest in cryo-ET is on the rise. As the pipeline matures, efforts will focus on streamlining procedures through the integration of a fully automated image processing pipeline, under development at the ESRF, optimizing the user experience, minimizing the waiting times between steps and expanding collaborations.

The SOS pipeline aims to provide SPA/cryo-ET infrastructure to users where this is not yet available. In this regard, a crucial upcoming initiative involves integrating ‘cryo-focused ion-beam scanning electron microscopy’ (cryoFIB-SEM) milling into the pipeline. This addition aims to streamline the workflow for cryo-ET experiments, allowing the preparation of lamellae and cryo-ET imaging at the same location. This not only ensures a comprehensive workflow but also simplifies the complexity associated with transporting lamellae. The integration of lamella preparation into the SOS pipeline will occur following the installation of the cryoFIB-SEM milling equipment at IBS-EM.
